# Software Application Profile: PXStools—an R package of tools for conducting exposure-wide analysis and deriving polyexposure risk scores

**DOI:** 10.1093/ije/dyac216

**Published:** 2022-11-16

**Authors:** Yixuan He, Chirag J Patel

**Affiliations:** Program in Bioinformatics and Integrative Genomics, Harvard Medical School, Boston, MA, USA; Department of Biomedical Informatics, Harvard Medical School, Boston, MA, USA; Department of Biomedical Informatics, Harvard Medical School, Boston, MA, USA

**Keywords:** Polyexposure risk score, PXS, exposure, environment, exposome, XWAS, risk score, machine learning, R package, software

## Abstract

**Motivation:**

Investigating the aggregate burden of environmental factors on human traits and diseases requires consideration of the entire ‘exposome’. However, current studies primarily focus on a single exposure or a handful of exposures at a time, without considering how multiple exposures may be simultaneously associated with each other or with the phenotype. Polyexposure risk scores (PXS) have been shown to predict and stratify risk for disease beyond or complementary to genetic and clinical risk. PXStools provides an analytical package to standardize exposome-wide studies as well as derive and validate polyexposure risk scores.

**Implementation:**

PXStools is a package for the statistical R.

**General features:**

The package allows users to (i) conduct exposure-wide association studies; (ii) derive and validate polyexposure risk scores with and without accounting for exposure interactions, using new approaches in regression modelling (hierarchical lasso);(iii) compare goodness of fit between models with and without multiple exposures; and (iv) visualize results. A data frame with a unique identifier, phenotype and exposures is needed as the only input. Various customizations are allowed including data preprocessing (removing missing or unwanted responses), covariates adjustment, multiple hypothesis correction and model specification (linear, logistic, survival).

**Availability:**

The PXStools source code is freely available on Github at [https://github.com/yixuanh/PXStools].

## Introduction

It has become increasingly clear that environmental exposures play important roles in human health. EXposome-wide association studies (XWAS) are agnostic tests of associations between exposures and phenotype. XWAS studies have previously identified numerous environmental exposures associated with diseases.^1–6^ Methods to conduct XWAS are available, such as the *rexposome* and *exposomeShiny* packages.^7^^,^^8^ However, a more comprehensive approach leveraging machine learning techniques, which captures the total contribution of many non-genetic exposures in a single value while considering dense inter-exposure correlation,^5^^,^^9^^,^^10^ is still elusive.

We recently introduced the polyexposure risk score (PXS) for type 2 diabetes, which summarizes the total estimated association of many independent exposures.^11^ Our method is based on a data-driven machine learning ‘feature selection’ procedure. Briefly, we used our approach first to associate over 100 exposure and lifestyle factors of participants in the UK Biobank cohort with incident type 2 diabetes (T2D). We then selected a non-redundant and statistically independent set of 12 exposures associated with incident T2D in an additional and non-overlapping cohort. The final PXS was a weighted sum of these exposures, and was able to improve the prediction and reclassification of incident T2D beyond or complementary to clinical factors and a genome-wide polygenic risk score.^11^

In this paper, we extend PXS methods and present the PXStools analytical package, a set of R-based functions to conduct exposure-related PXS-related analysis. The package contains functions that allow users to conduct XWAS, derive and validate PXS, assess improvement of fit between models (e.g. with and without PXS) and visualize results for any set of exposures and phenotypes. The package accommodates a number of ‘regularized’ regression and non-linear approaches for real values and binary or time-to-event outcomes. The tools documented here will help accelerate the evaluation of PXS in other disease outcomes and other research contexts.

## Implementation

The package and simulated datasets can be downloaded from [https://github.com/yixuanh/PXStools]. A description of the simulated dataset can be found in Supplementary File Section S1 (available as Supplementary data at *IJE* online). There are five functions: XWAS(), plot_coeff_xwas(), manhattan_xwas(), PXSgl() and PXS(). In summary, XWAS() conducts exposure-wide regression, manhattan_xwas() and plot_coeff_xwas() are visualization tools of the *P*-value and the effect sizes from the XWAS() output, respectively, PXSgl() and PXS() calculate PXS with and without including pair-wise interactions and delta_pred() evaluates the difference in goodness of fit between two models via bootstrapping. Tutorials for implementing each function are available at [https://github.com/yixuanh/PXStools/blob/main/README.md].

The input data frame must contain the following columns: ‘ID’ for the unique identifiers of individuals in the data frame, ‘PHENO’ for the phenotype of interest (binary or continuous) and ‘TIME’ for time-to-event or censoring if running survival analysis. In XWAS(), PXS() and PXSgl() functions, the user can input any set of exposures of interest and designate the regression approach, including ‘lm’ for linear models (real-values phenotypes), ‘logistic’ for logistic model (binary phenotypes) and ‘cox’ for Cox proportional hazards regression (time-to-event analysis). Certain functions allow for the option to save intermediate files. For XWAS(), the intermediate file contains the effect sizes of the covariates in every model. For PXS(), the intermediate file contains the results from the regularization step as well the coefficients of every factor in the final multivariable model. For the PXS() and PXSgl() functions, identification numbers for three non-overlapping ‘sets’ of individuals are to be input: groups A, B and C. Group A and B are used for variable selection and model calibration, respectively. PXSs will be generated for individuals in Group C only. The user can also assign a set of covariates to adjust for, as well as which exposure factors to remove, a priori from the analysis.

The XWAS() function performs exposure-wide association study.^1^ The default method to correct for multiple hypothesis testing is the Benjamini and Yekutieli method.^12^ The function runs as follows:

for each exposure:

  remove individuals with missing or unwanted exposure response;

  regress phenotype to exposure;

  store *P*-value and association/effect size from summary statistics;

end:

correct for multiple comparison;

return summary statistics and false-discovery rate (FDR)-corrected *P*-value for all exposures.

The PXS() function performs variable selection via shrinkage and iterative stepwise regressions, and the risk score is a weighted sum of the selected independent exposures. Whereas many selection procedures are possible, we implemented a method that favours model simplicity to maximize interpretability. More specifically, we first use the glmnet package to conduct cross-validation regularization.^13^ Next, we perform stepwise selection on the non-zero exposures with a *P*-value threshold to obtain a much smaller, but independent, set of exposures associated with the phenotype. PXS() only considers main (non-interacting) effects in the model. The PXS() function runs as follows.

Step 1 (run on subset Group A):

- subset group A and input exposures (that passed some FDR threshold, for example);
- remove individuals with missing exposure responses (complete cases);
- run k-fold cross-validation for glmnet;
- store fitted glmnet object with ‘lambda’ that gives minimum mean cross-validated error.

Step 2 (run on subset Group B)*:*

- subset Group B and stored exposures from Step 1;
- remove individuals with missing or unwanted exposure responses;
- initiate backward selection with the output from Step 1;
- iteratively removed non-significant variables until only independently significant variables (at *P* <0.05) remain;
- store final model.

Step 3 (predict in subset Group C):

- subset roup C;
- predict PXS using the stored model from Step 2;
- assess prediction accuracy in Group C.

As an alternative, PXSgl() considers pairwise interactions between exposure terms using hierarchical group-lasso regularization, as implemented through the glinternet package.^14^ This method favours a strong ‘hierarchy’: when an interaction is estimated to be non-zero, both of the terms are considered as main effects in the model. Since glinternet constructs only linear and logistic models, we recalibrate the models in a held-out cohort to accommodate for survival analysis.^15^ PXSgl() runs as follows.

Step 1 (run on subset Group A)*:*

- subset group A and input exposures;
- remove individuals with missing or unwanted exposure responses;
- run k-fold cross-validation for glinternet;
- if ‘survival’ model, run group lasso as ‘logistic’ model;
- store fitted glinternet object with ‘lambda’ that gives minimum mean cross-validated error.

Step 2 (run on subset Group B):

- subset sample for group B and stored exposures from Step 1;
- remove individuals with missing or unwanted exposure responses;
- recalibrate model with selected predictors to match user designated regression;
- store final model.

Step 3 (predict in subsetGgroup C):

- subset Group C;
- predict PXS using the stored model from Step 2;
- assess prediction accuracy in group C.

Our procedure accommodates LASSO (by default), ridge or elastic net regulation methods. The elastic penalty is controlled by a single alpha value. Setting alpha to 1 implements LASSO (by default), alpha = 0 implements ridge and alpha between 0 and 1 implements elastic net. The best lambda values in PXS() and PXSgl() are both determined through k-fold cross-validation. To minimize the loss of samples used in optimizing the lambda parameter, we recommend using only significant exposure from the XWAS() step as exposure inputs into PXS() and PXSgl(). We combined both regularization and stepwise selection in our PXS method in order to maximize interpretability and minimize the complexity of the final model. Using either method alone resulted in a large set of exposures, some of which may not be significantly associated with the outcome in the final multivariable model (e.g. did not have independent associations). Our method reduces the number of exposures included in the model without compromising prediction accuracy, and furthermore, constructs a PXS that ensures that variables are independent of each other in their contribution to the additive risk or correlation with a phenotype. Finally, all exposures are significantly associated with the outcome in the final multivariable model.

The delta_pred() function assesses the goodness of fit of two models separately and then uses bootstrap analysis to derive a difference in prediction accuracy between the two models (e.g. one with the PXS and one without). The goodness of fit is evaluated by R^2^ for linear regression models, area under the curve (AUC) for logistic regression models and Harrell’s concordance index (C-index) for Cox regression models.

## Use

To demonstrate the use and utility of PXStools, we derived and validated polyexposure risk scores (PXS) for 12 incident diseases and quantitative phenotypes in the UK Biobank^16^: atrial fibrillation (AF), coronary artery disease (CAD), chronic obstructive pulmonary disease (COPD), type 2 diabetes (T2D), height, systolic blood pressure, body mass index (BMI), high-density lipoprotein (HDL), total cholesterol, triglycerides and forced expiratory volume 1 s (FEV-1) (see Supplementary File Section S2, available as Supplementary data at *IJE* online for detailed phenotype classification). For the four disease phenotypes, we defined cases as individuals who at the time of first assessment did not have the disease but were subsequently diagnosed. All other continuous phenotypes are cross-sectional measurements at the first time of assessment. We used an initial set of 98 543 (Group A) and 98 495 (Group B) individuals to train and calibrate the model, respectively. The initial set of exposures contained 109 variables of physiological state, environmental exposure, and self-reported behaviour collected during the first assessment visit period (see Supplementary File Section S3, available as Supplementary data at *IJE* online for detailed descriptions of exposures). In our analysis, we used all default parameters prespecified in our code. The run time for each phenotype was within 1–2 min for the XWAS procedure and <5 min for the PXS procedure on an 8-core system and 100 GB memory on the ‘Orchestra 2’ (O2) high performance computing cluster, supported by the Research Computing Group, at Harvard Medical School. See [https://it.hms.harvard.edu/our-services/research-computing] for more information (Supplementary File Section S4, available as Supplementary data at *IJE* online). The exposures selected in the final PXS for each phenotype are shown in Supplementary File Section S5 (available as Supplementary data at *IJE* online).

The most common exposure selected across all phenotypes after our procedure included: employment status, which was selected in all 12/12 phenotype PXSs; physical activity and home homeownership status were in 11/12 phenotype PXSs; smoking status, TV time and qualifications were in 10/12 phenotypes PXSs. The least common exposures were nitrogen dioxide (NO_2_) and particulate matter <2.5 μm or less (PM_2.5_) levels, which were in only one phenotype PXS. BMI and FEV-1 had the most variables in their PXS, with 33 and 19 exposures, respectively. Incident CAD and AF had the fewest variables in their PXS, with only five and six exposures, respectively.

**Figure 1:**
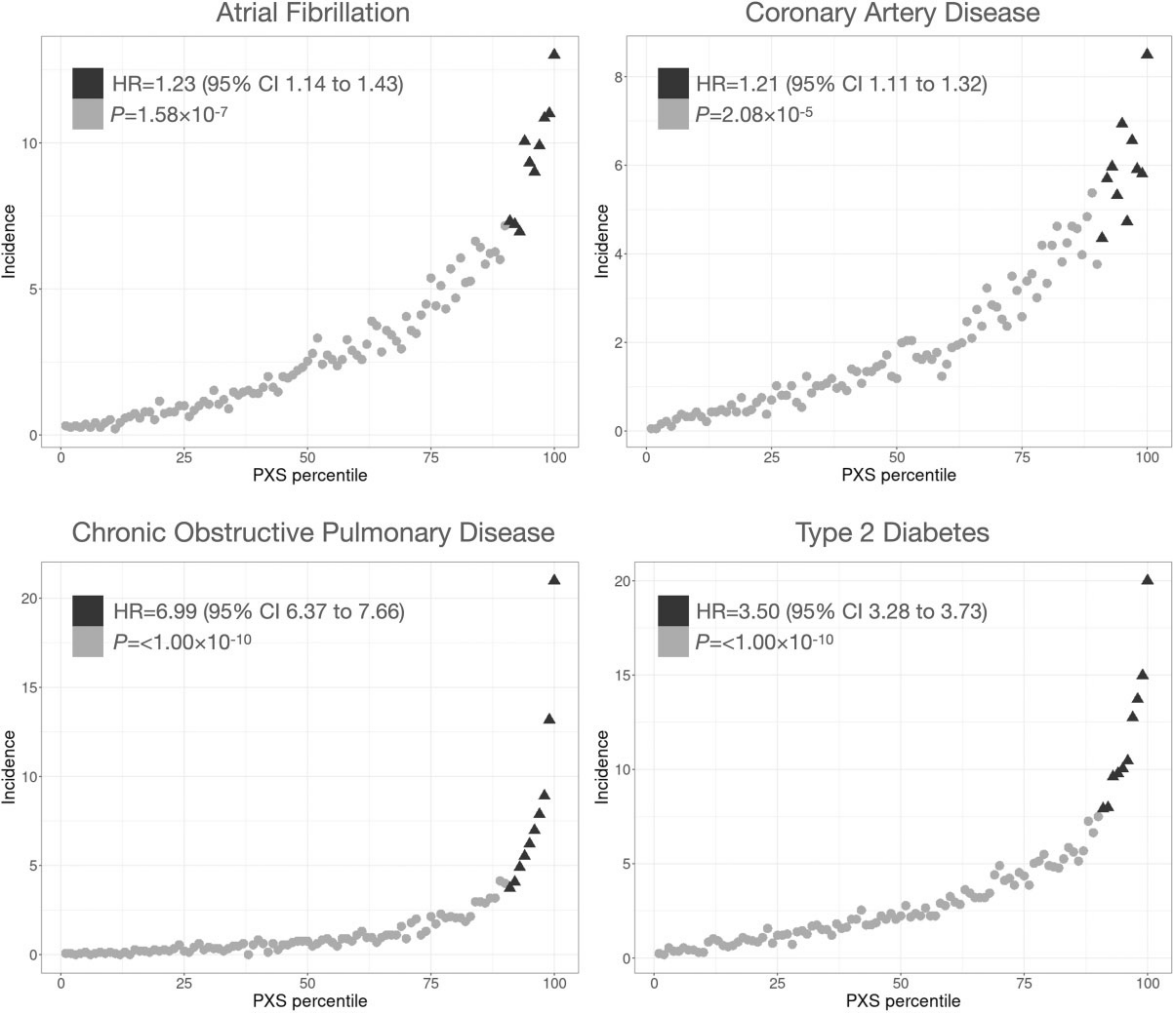
Risk stratification of four diseases based on PXS percentiles. Individuals were binned into 100 groups based on their PXS percentiles. The incidence of each disease within each bin is calculated and plotted on the y-axis. The top decile of PXS values for each disease is represented by black triangles, and the remaining population is represented by grey circles. The HR of the top decile versus the remaining population for each disease is shown. Note the incidence ranges vary between panels. HR, hazard ratio; PXS, polyexposure risk score

We next demonstrated the clinical utility of PXS in the four incident disease phenotypes: AF, CAD, COPD and T2D. Figure 1 demonstrates risk stratification by PXS in all four diseases. Furthermore, individuals in the top decile of PXS (labelled blue in Figure 1) have higher risks of disease, as defined by the hazard ratio (HR), than the remaining individuals (labelled orange in Figure 1): HR = 1.23 (95% CI 1.14 to 1.32, *P *=* *1.58 x 10^-7^) for AF, HR* *=* *1.21 (95% CI 1.11 to 1.32, *P *=* *2.08 x 10^-5^) for CAD, HR = 6.99 (95% CI 6.37 to 7.66, *P *=* *<1.00 x 10^-10^) for COPD and HR = 3.50 (95% CI 3.28 to 3.73, *P *=* *<1.00 x 10^-10^) for T2D. The PXS for COPD had the greatest predictive power, with a C-index of 0.828 (95% CI 0.820 to 0.836) (Table 1; and see Supplementary File Section S6, available as Supplementary data at *IJE* online for a description of models). Since smoking status was an exposure variable in all four disease phenotypes and is an established risk factor for disease and mortality, we compared the predictive power of the PXS with smoking alone. PXS tentatively had higher predictive power than smoking alone. For example, in T2D the C-index was 0.611 (95% CI 0.654 to 0.668) for the model with baseline covariates only, 0.672 (95% CI 0.665 to 0.679) with smoking alone and 0.751 (95% CI 0.745 to 0.757) for the full model. Smoking status (current and past smokers versus non-smokers) also identified individuals with high risks for the four diseases (Supplementary File Section S7, available as Supplementary data at *IJE* online). For example, compared with non-smokers, current smokers had an HR = 1.85 (95%CI 1.70 to 2.00, *P *=* *<1.00 x 10^-10^) and HR = 3.33 (95% CI 2.98 to 3.73, *P *=* *<1.00 x 10^-10^) for T2D and COPD, respectively. Compared with non-smokers, previous smokers had an HR = 1.47 (95% CI 1.49 to 1.56, *P *=* *<1.00 x 10^-10^) and HR = 13.42 (95% CI 11.94 to 15.08, *P *=* *<1.00 x 10^-10^) for T2D and COPD, respectively.

**Table 1:** The first two columns are the UK Biobank identification numbers and names of the 43 exposures that have been selected in any of the 12 phenotypes. A 1 in each column indicates that the variable is selected for that trait’s polyexposure risk scores (PXS, shaded cells); a 0 means it is not. The total number of exposures selected for each trait is shown in the last row. The number of phenotype PXSs that incorporate each exposure is shown in the last column

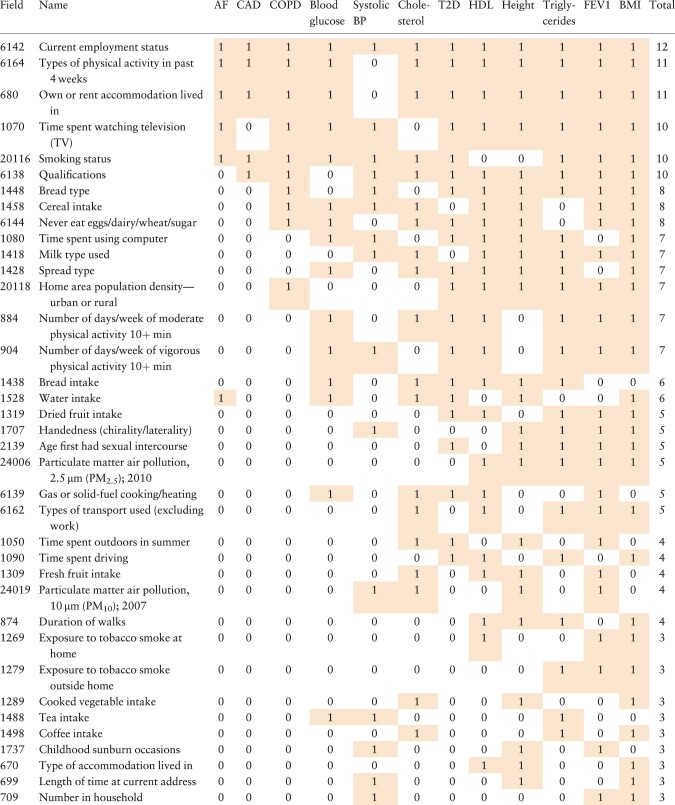														

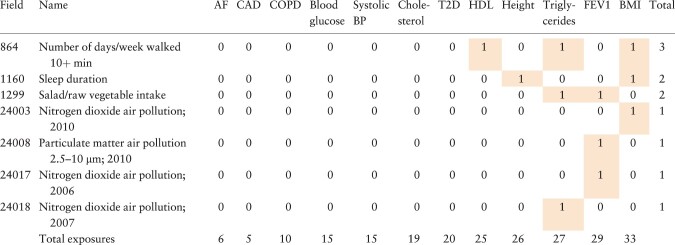														

PXS, polyexposure risk score; AF, atrial fibrillation; CAD, coronary artery disease; COPD, chronic obstructive pulmonary disease; T2D, type 2 diabetes; HDL, high-density lipoprotein; BP, blood pressure; FEV1, forced expiratory volume, 1 s; BMI body mass index.

Covariates, such as sex and age, are used to address potential measured confounding in XWAS. However, their relationship and confounding characteristics may differ between different exposures. We compared the association sizes of sex and age covariates in the XWAS analysis of all four diseases (Supplementary File Section S8, available as Supplementary data at *IJE* online). For all four diseases, being male (compared with being female) and being older conferred greater risk in all univariate associations between exposures and disease. The effect of sex was the strongest in CAD and weakest in COPD. The effect of age was the strongest in T2D and weakest in AF (Supplementary File Section S8a).

We also calculated the PXS for T2D using the group lasso PXSgl() method to consider interactions between exposures. Much greater computational power and run time were required, as all pairwise interactions are considered by the procedure. The final PXS consisted of a larger set of exposures but had a lower predictive power (C-index of 0.7, 95% CI 0.692 to 0.708) (Supplementary File Section S9, available as Supplementary data at *IJE* online). There were 37 main effects including covariates (sex, age, assessment centre and the first four principal components) and 37 interaction pairs in the final model. We hypothesize that PXSgl() may be more prone to overfitting as there is no additional stepwise selection procedure. For T2D, using the LASSO-based PXS() method resulted in a PXS composed of 20 exposure factors, and the PXSg() method resulted in a PXS composed of 74 total variables. We calculated the false-positive and false-negative rates using both PXS() or PXSgl() methods to derive T2D PXS, and found them to be comparable (Supplementary File Section S10, available as Supplementary data at *IJE* online).

## Discussion

PXStools is an analytical toolbox aimed to help researchers estimate polyexposure risk scores, an aggregate score that summarizes the non-genetic measured risk for an incident phenotype or correlation with a cross-sectional phenotype. The package accommodates three types of regression models and is adaptable to user customizations. To minimize loss of sample and maximize interpretation, our methods use both a simple univariate and an iterative regularized selection procedure to build PXSs.

We include two methods to calculate PXS in our package—PXS(), an iterative regularization and stepwise selection-based procedure, and PXSgl(), a group LASSO-based procedure that considers pairwise exposure interactions. We caution users in choosing the PXSgl() method as it is much more computationally intensive.

As an application of the package, we calculated the PXS for four different incident diseases and eight continuous phenotypes. We demonstrated the simplicity and efficiency of our method for various types of models. The PXS is easily interpretable and able to stratify the risk of disease. Our package has broad applications since the software can be easily deployed for any set of exposures and phenotypes.

Despite the importance of many exposures in phenotypic variation, most studies have investigated single exposures in isolation. Our PXStools package supports multiple methods to investigate the cumulative association of the exposome—the combination of many environmental factors.^17^ We introduce methods to consider exposures in an additive fashion and through pairwise interactions, favouring model interpretability and minimizing model complexity. As non-genetic exposure variables may be correlated with one another,^5^^,^^18^ we desired to estimate the independent associations between the exposures and outcome estimation of the additive PXS. We also can consider distributions of covariate association sizes to assess the robustness of exposure-disease associations.^19^ We recognize that a limitation of our package is that it is unable to consider higher orders of exposure interactions (e.g. three-way). We also relied on pre-processing heuristics, the PHEASANT software tool, to transform all variables.^20^

We claim that the package has broad applications, such as comparing the PXS against single exposures in risk for outcomes, increasing power for gene-by-environment studies and developing screening tools to reclassify individuals. More importantly, we hope that our package will help enhance replicable research for exposure- and exposome-related analysis.

## Ethics approval

UK Biobank data was accessed under application number 22881. All participants from the UK Biobank provided written informed consent for anonymized data to be used for research and publication.^16^

## Supplementary Material

dyac216_Supplementary_Data

## Data Availability

PXStools can be downloaded from [https://github.com/yixuanh/PXStools]. All simulated datasets are also available through the GitHub page. UK Biobank data are obtained through the process described at [https://www.ukbiobank.ac.uk/principles-of-access/].
