# Supplementary material for: Software Application Profile: PXStools—an R package of tools for conducting exposure-wide analysis and deriving polyexposure risk scores
Source: Int J Epidemiol. 2022 Nov 16;52(2):633–40. doi: 10.1093/ije/dyac216 (PMC10114106; doi:10.1093/ije/dyac216)
Supplement: dyac216_Supplementary_Data [file dyac216_supplementary_data.docx]

# Supplementary File

# Section S1: simulated dataset

We randomly generated the quantitative phenotype using the baseR rnorm() function with mean of 100 and standard deviation of 15. The simulated toy dataset contains 5000 artificially generated individuals with columns for individual IDs, randomly generated sex, gender, continuous and categorical variables, and a continuous (CONTINUOUS) or binary (BINARY) phenotype. The BINARY toy dataset also contains a randomly generated TIME column for survival analysis. We derived the binary phenotype by rounding the ranked values of the quantitative phenotype. Sex was randomly assigned with 1:1 ratio, age was randomly generated with mean 50 and standard deviation of 10. The exposure variables have random amounts of correlation with each other and with the phenotype. Numerical exposure variables are assigned a random amount of correlation (between 0 and 1, average correlation of 0.186) with the continuous phenotype. The continuous exposures have randomly generated means between -5 and 5 and randomly generated standard deviations between 0 and 3. Categorical variables are randomly generated letters with a random amount of unique values.

#

# Section S2: Disease phenotype classification

We classified diseases based on self-report, linked hospital admission records for International Classification of Disease (ICD-9 and ICD-10) codes and Office of Population Census and Surveys (OPCS-4) codes. Atrial fibrillation (AF) cases were defined as having an ICD-9 code of 427, ICD-10 code of I48.X, OPCS-4 code of K57.1, K62.X, or having self-reported AF in an interview. Coronary artery disease (CAD) cases were defined as having an ICD-9 code of 410, 411, or 412, ICD-10 code of I21.X, I22.X, I23.X, I24.1, or I25.2, OPCS-4 code of K40.1-40.4, K41.1-41.4, K45.1-45.5, K49.1, K49.2, K49.8, K49.9, K50.2, K75.1-75.4 or K75.8-75.9, or having self-reported CAD in an interview. Chronic obstructive pulmonary disease (COPD) cases were defined as having an ICD-9 code of 490, 491, 492, 494, ICD-10 code of J41.X, J43.X, J44.X, J98.2, J98.3, or having self-reported COPD in an interview. Type 2 diabetes (T2D) cases were defined as having an ICD-10 code of E11.X, ICD-9 of 250, or having self-reported T2D in an interview. We calculated time to event by taking the difference between the first assessment when exposures were measured to the time of first diagnosis. We derived censoring time based on an individual’s assessment center, and we obtained the date of death from linked death registry data.

# Section S3: exposure classification

We defined the initial set of exposure variables as indicators of physiological state, environmental exposure, and self-reported behavior collected during the first assessment visit period (2006-2010). We first extracted all variables in the categories ‘Reception’, ‘Employment’, ‘Sociodemographics’, ‘Lifestyle and environment’, ‘Estimated nutrients yesterday’, ‘Early life factors’, ‘Typical diet yesterday’, ‘Meal type yesterday’, ‘Spreads/sauces/cooking oils yesterday’, ‘Alcoholic beverages yesterday’, ‘Hot/cold beverages yesterday’, ‘Cereal yesterday’, ‘Milk/eggs/cheese yesterday’, ‘Bread/pasta/rice yesterday’, ‘Soup/snacks/pastries yesterday’, ‘Meat/fish yesterday’, ‘Milk/eggs/cheese yesterday’, ‘Vegetarian alternatives yesterday’, ‘Fruit/vegetables yesterday’, ‘Residential air pollution’, ‘Residential noise pollution’, resulting in 519 unique variables in total. Of these, we only considered variables that had less than 10% missingness, resulting in 111 variables remaining exposures.

To process the exposure data, we used the PHESANT software tool [(21)](https://www.zotero.org/google-docs/?6JBtU6). In summary, PHESANT assigns one of four data types to UK Biobank data: continuous, ordered categorical, unordered categorical, and binary. We removed responses with negative-encoded values (such as ‘Prefer not to answer’ and ‘Do not know’). Continuous variables were transformed to a normal distribution using an inverse normal rank transformation. In cases where the variable cannot be transformed due to a large number of participants with the same value (e.g., rank order variables), the variables are encoded as an ordered categorical variable with three roughly equal categories. For unordered categorical variables, the response with the largest number of participants was selected as the reference group.

# Section S4: Run time for eXposure wide association study (XWAS) analysis and polyexposure risk score (PXS) construction. All calculations were run with an 8 core system and 100 GB memory on the Harvard O2 computing platform. AF: atrial fibrillation, CAD: coronary artery disease, COPD: chronic obstructive pulmonary disease, T2D: type 2 diabetes, BMI: body mass index, HDL: high-density lipoprotein, BP: blood pressure, FEV1: force expiratory volume,1 second.

| **Phenotype** | **XWAS (seconds)** | **PXS (seconds)** |
| --- | --- | --- |
| AF | 94.87 | 114.36 |
| CAD | 94.05 | 84.82 |
| COPD | 95.05 | 274.37 |
| T2D | 93.78 | 220.88 |
| height | 32.38 | 37.49 |
| Systolic BP | 32.33 | 27.95 |
| BMI | 42.62 | 42.62 |
| Blood glucose | 35.33 | 28.59 |
| HDL | 34.82 | 32.79 |
| Cholesterol | 34.33 | 37.51 |
| Triglycerides | 35.91 | 32.56 |
| FEV1 | 38.18 | 42.77 |

#

#

# Section S5: Predictive ability of PXS for each disease and trait.

C indices are shown for disease phenotypes and R^2’^’s are shown for real-valued traits. The reduced model includes baseline covariates of sex, age, first four principal components of ancestry, and assessment center. The smoking model includes all baseline covariates and smoking status. The full model also includes baseline covariates and PXS. The change in predictive ability of the full versus the reduced model is shown in the last column. C-index: concordance index, CI: confidence interval, SD: standard deviation, AF: atrial fibrillation; CAD: coronary artery disease; COPD: chronic obstructive pulmonary disease; T2D: type 2 diabetes, BMI: body mass index, HDL: high density lipoprotein; FEV1: forced expiratory volume, 1-second.

|  |  | **Reduced model** | | **Smoking model** | | **Full model** | | **Δ prediction (Full-Reduced)** | |
| --- | --- | --- | --- | --- | --- | --- | --- | --- | --- |
| **Phenotype** | **Cases/N** | **C index** | **95 % CI** | **C index** | **95 % CI** | **C index** | **95 % CI** | **C index** | **95 % CI** |
| AF | 5,964/189,849 | 0.733 | 0.727–0.739 | 0.739 | 0.730–0.741 | 0.742 | 0.736–0.748 | 0.009 | 0.007– 0.010 |
| CAD | 3,990/185,980 | 0.722 | 0.715–0.729 | 0.731 | 0.724–0.738 | 0.737 | 0.703–0.744 | 0.015 | 0.011–0.018 |
| COPD | 2,382/144,867 | 0.690 | 0.680–0.700 | 0.797 | 0.788–0.806 | 0.828 | 0.820–0.836 | 0.137 | 0.128–0.147 |
| T2D | 673/165,461 | 0.661 | 0.654–0.668 | 0.672 | 0.665-0.679 | 0.751 | 0.745–0.757 | 0.09 | 0.084 –0.096 |
|  | **Mean (SD)** | **R^2^** | **95 % CI** |  |  | **R^2^** | **95 % CI** | **R^2^** | **95 % CI** |
| Height (cm) | 169.16 (9.19) | 0.534 | 0.531–0.537 |  |  | 0.551 | 0.548–0.554 | 0.017 | 0.016–0.018 |
| Systolic BP (mmHg) | 140.11 (19.46) | 0.123 | 0.12–0.126 |  |  | 0.135 | 0.133–0.138 | 0.012 | 0.011–0.013 |
| BMI (Kg/m^2^) | 27.33 (4.61) | 0.011 | 0.01–0.013 |  |  | 0.135 | 0.131–0.139 | 0.124 | 0.121–0.127 |
| Blood glucose (mmol/L) | 5.1 (1.16) | 0.017 | 0.015–0.018 |  |  | 0.030 | 0.028–0.032 | 0.013 | 0.012–0.014 |
| HDL (mmol/L) | 1.47 (0.38) | 0.179 | 0.175–0.183 |  |  | 0.224 | 0.220–0.229 | 0.045 | 0.043–0.047 |
| Cholesterol (mmol/L) | 5.74 (1.13) | 0.031 | 0.029–0.033 |  |  | 0.051 | 0.049–0.054 | 0.021 | 0.019–0.022 |
| Triglycerides (mmol/L) | 1.73 (1) | 0.052 | 0.049–0.054 |  |  | 0.084 | 0.081–0.086 | 0.032 | 0.030–0.034 |
| FEV1 (L) | 2.89 (0.78) | 0.491 | 0.487–0.495 |  |  | 0.507 | 0.502–0.510 | 0.015 | 0.014–0.017 |

# Section S6: Statistical analysis

We constructed a baseline model with just covariates sex, age, principal component (PC)1-4, and assessment center, then we added either employment status. We assessed the prediction of each model using Harrell’s C-index for disease phenotypes and R^2^ for continuous phenotypes. For disease phenotypes, we calculated the hazard ratio by taking the exponent of the coefficient in the model. The p values are those of the hazard ratio, *ie* the p-values from ​​testing the null hypothesis that the hazard ratio is 1. 95% percentile bootstrap confidence intervals with 100 bootstrap samples were derived with the boot package in R. Cox regression was implemented by the survival package in R.

# Section S7: Hazard ratio for smoking status (versus never smokers) in disease phenotypes. All associations were significant (*P*<0.001). HR: hazard ratio. CI; confidence interval, AF: atrial fibrillation; CAD: coronary artery disease; COPD: chronic obstructive pulmonary disease; T2D: type 2 diabetes.

|  | **Previous smokers** | | **Current smokers** | |
| --- | --- | --- | --- | --- |
| **Phenotype** | **HR** | **95 % CI** | **HR** | **95 % CI** |
| AF | 1.2646 | 1.1976–1.335 | 1.3531 | 1.1294–1.4770 |
| CAD | 1.1278 | 1.1372–1.3042 | 2.0182 | 1.840–2.2124 |
| COPD | 3.3312 | 2.9979–3.7263 | 13.4213 | 11.9447–15.0803 |
| T2D | 1.4704 | 1.3893–1.5563 | 1.8452 | 1.6991–2.0037 |

# Section S8: Adjustment effect of covariates in XWAS analysis. We plot the adjustment effects of sex (A.) and year of birth (B.) in all XWAS analyses (N=113) for each of the four disease phenotypes. Being male conferred greater risk (A.) and being born later conferred protection (B.) for all four diseases. AF: atrial fibrillation; CAD: coronary artery disease; COPD: chronic obstructive pulmonary disease; T2D: type 2 diabetes; HR: hazard ratio.


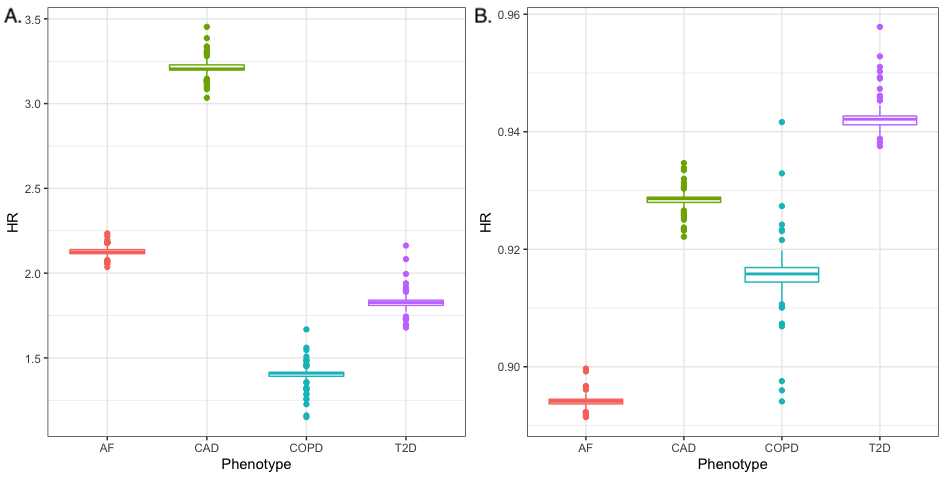


#

# Section S9: Group LASSO PXS for T2D

We ran the PXSgl algorithm for T2D with 5000 individuals for training and 59 input exposures that were XWAS hits. The run time was 3760.52 seconds. The main effects were: Sex, Year of birth, principa components (PC) 1-4, assessment center, Time spend outdoors in summer, Time spent outdoors in winter, Time spent watching television (TV), Time spent driving, Sleeplessness / insomnia, Snoring, Daytime dozing / sleeping (narcolepsy), Smoking/smokers in household, Exposure to tobacco smoke at home, Bread type, Cereal intake, Hot drink temperature, Water intake, Alcohol intake frequency., Comparative body size at age 10, Facial ageing, Maternal smoking around birth, Smoking status, Age first had sexual intercourse, Plays computer games, Use of sun/UV protection, Nitrogen oxides air pollution; 2010, Particulate matter air pollution (pm10); 2010, Nitrogen dioxide air pollution; 2007, Current employment status, Never eat eggs, dairy, wheat, sugar, Types of physical activity in last 4 weeks, Number of days/week walked 10+ minutes, Number of days/week of moderate physical activity 10+ minutes, Number of days/week of vigorous physical activity 10+ minutes, and Usual walking pace. The interaction terms were:

| **Term 1** | **Term 2** | **Date Type** |
| --- | --- | --- |
| Number of days/week of moderate physical activity 10+ minutes | Time spend outdoors in summer | Continuous x Continuous |
| Number of days/week walked 10+ minutes | Time spent watching television (TV) | Continuous x Continuous |
| Birth year | Time spent driving | Continuous x Continuous |
| pc2 | Time spent driving | Continuous x Continuous |
| assesment_center | Exposure to tobacco smoke at home | Continuous x Continuous |
| Water intake | Age first had sexual intercourse | Continuous x Continuous |
| Time spent driving | Nitrogen oxides air pollution; 2010 | Continuous x Continuous |
| Time spent driving | Particulate matter air pollution (pm10); 2010 | Continuous x Continuous |
| Age first had sexual intercourse | Nitrogen dioxide air pollution; 2007 | Continuous x Continuous |
| Particulate matter air pollution (pm10); 2010 | Nitrogen dioxide air pollution; 2007 | Continuous x Continuous |
| Birth year | Number of days/week of moderate physical activity 10+ minutes | Continuous x Continuous |
| pc4 | Number of days/week of moderate physical activity 10+ minutes | Continuous x Continuous |
| pc3 | Number of days/week of vigorous physical activity 10+ minutes | Continuous x Continuous |
| Snoring | Time spend outdoors in summer | Categorical x Continuous |
| Alcohol intake frequency. | Time spent outdoors in winter | Categorical x Continuous |
| Sleeplessness / insomnia | Time spent driving | Categorical x Continuous |
| Plays computer games | Time spent driving | Categorical x Continuous |
| Sleeplessness / insomnia | Exposure to tobacco smoke at home | Categorical x Continuous |
| Facial ageing | Cereal intake | Categorical x Continuous |
| Sleeplessness / insomnia | Particulate matter air pollution (pm10); 2010 | Categorical x Continuous |
| Hot drink temperature | Birth year | Categorical x Continuous |
| Snoring | Number of days/week of moderate physical activity 10+ minutes | Categorical x Continuous |
| Sex | Number of days/week of moderate physical activity 10+ minutes | Categorical x Continuous |
| Hot drink temperature | Number of days/week of vigorous physical activity 10+ minutes | Categorical x Continuous |
| Current employment status | pc4 | Categorical x Continuous |
| Snoring | Bread type | Categorical x Categorical |
| Sex | Comparative body size at age 10 | Categorical x Categorical |
| Never eat eggs, dairy, wheat, sugar | Comparative body size at age 10 | Categorical x Categorical |
| Daytime dozing / sleeping (narcolepsy) | Maternal smoking around birth | Categorical x Categorical |
| Smoking/smokers in household | Smoking status | Categorical x Categorical |
| Sex | Smoking status | Categorical x Categorical |
| Daytime dozing / sleeping (narcolepsy) | Plays computer games | Categorical x Categorical |
| Sleeplessness / insomnia | Use of sun/uv protection | Categorical x Categorical |
| Usual walking pace | Never eat eggs, dairy, wheat, sugar | Categorical x Categorical |
| Daytime dozing / sleeping (narcolepsy) | Types of physical activity in last 4 weeks | Categorical x Categorical |
| Sex | Types of physical activity in last 4 weeks | Categorical x Categorical |
| Sex | Usual walking pace | Categorical x Categorical |

**Section S10: False positive and false negative rates for T2D PXS.**

We calculated the false positive and false negative rates for T2D using PXS() and PXSgl() algorithms using a range of PXS percentiles as risk thresholds. T2D: type 2 diabetes.

|  | False Positive | | False negative | |
| --- | --- | --- | --- | --- |
| Risk Threshold | PXS() | PXSgl() | PXS() | PXSgl() |
| 99% | 0.102 | 0.105 | 0.898 | 0.895 |
| 98% | 0.142 | 0.147 | 0.858 | 0.853 |
| 95% | 0.239 | 0.249 | 0.761 | 0.751 |
| 90% | 0.364 | 0.375 | 0.636 | 0.625 |
| 80% | 0.527 | 0.543 | 0.473 | 0.457 |
| 70% | 0.660 | 0.667 | 0.340 | 0.333 |
| 60% | 0.758 | 0.769 | 0.242 | 0.231 |
